# Effect of stocking density and different diets on growth of Percula Clownfish, *Amphiprion percula* (Lacepede, 1802)

**DOI:** 10.1186/s40064-015-0967-x

**Published:** 2015-04-16

**Authors:** João Chambel, Vera Severiano, Teresa Baptista, Susana Mendes, Rui Pedrosa

**Affiliations:** MARE – Marine and Environmental Sciences Centre, ESTM, Instituto Politécnico de Leiria, 2520-641 Peniche, Portugal

**Keywords:** *Amphiprion percula*, Anemonefishes, Stocking density, Nutrition, Ornamental fish

## Abstract

The aim of this study was to evaluate the influence of stocking density (0.5, 1, 2 and 3 fishL^−1^) and commercial marine fish diets (diet A, B, C and D) over four months on specific growth rate, condition factor, percentage without anomalous pigmentation (partial or total lack of white bands -miss-band) and survival of juvenile *Amphiprion percula*.

Results showed that at 0.5 fishL^−1^ densities induced the best survival (100%) and also the maximum percentage of fish without miss-band (58.33 +/−4.417%). The maximum SGR was obtained for the 0.5 fishL^−1^ (0.459 ± 0.023% cm/day). However, the best condition factor (2.53 +/− 0.27) was achieved for 2 fishL^−1^ densities. There were no significant differences in survival (68.9 to 84.5%), fish without miss-bands (18.03 to 26.92%) and condition factor (1.92 to 2.1) among diets during the experimental period. On the other hand, diet C (with 41% crude protein) supported the best SGR (0.485 ± 0.001% cmday^−1^).

The results suggested that stocking density are critical and more relevant when compared with the different diet tested, namely on specific growth rate, condition factor, the miss-band and survival of juvenile percula clownfish.

This study has particular significance with regards to anemonefishes husbandry in terms of survival and production efficiency.

## Introduction

The marine ornamental industry has grown in last years. However is projected that less than 10% of marine animals sell in the world are originate from captive production. During last decades the unsustainable capture of wild organisms to the ornamental trade have serious consequences with the degradation of habitats and populations of tropical and subtropical marine fish are being depleted worldwide to supply increasing demands of the aquarium industry (Moorhead and Zeng 2010; Chambel et al. 2013; Araujo et al. 2014). In line with this view, it has been recommended the use of specimens produced in captivity. This approach reduces the utilization of natural resources, is therefore substantially more sustainable than relying on wild-caught organisms and may even be used to restore depleted ornamental populations (Ziemann 2001). One of the main problem with the production of marine ornamental species is optimize production in order to compete with less expensive specimens collected from the wild (Calado 2006; Jamabo and Keremah 2009; Chambel et al. 2014). The anemonefish, *Amphiprion percula* is a tropical coral reef fish belonging to the family Pomacentridae fishes and anemones and sub family Amphiprioninae. Are found in warmer waters in the Pacific Ocean, Indian Ocean, off northwest Australia, southeast Asia and Japan and they are one of the most popular marine ornamental fish (Dhaneesh et al. 2012b). This species, as other of *Amphiprion* species, is of the few marine ornamental species produced in captivity, unfortunately, anomalous pigmentation, consisting of partial or total lack of white bands (miss-band) is a common problem in the production process of this species (Avella et al. 2010). Pigmentation abnormalities reduce the market of fish so, solve this problem represent an important challenge to the ornamental aquaculture industry (Copeman and Parrish 2002).

Diet and stocking densities are two major factors in aquaculture influencing growth, welfare, and health (Ellis et al. 2002; Alcorn et al. 2003). Feed costs make up a large part of the total fish production costs. The optimization of growth rates and feed efficiency depends on the quantity of food delivered, feeding method and feeding frequency, quality and composition of the diet (Gélineau et al. 1998; Yang et al. 2003; Erondu et al. 2006).

Stocking density directly influences survival, growth, behaviour, water quality and feeding. In aquaculture, stocking density is the concentration which fish are stocked into a system (Gomes et al. 2006; de Oliveira et al. 2012). Generally, increases in stocking density results in directly increase on the stress condition, causing a reduction in growth rate and food utilization (Sharma and Chakrabarti 1998). On the other hand, in very low densities, fishes may not form shoals and may feel unprotected.

Consequently, identifying the optimum stocking density for a species is a critical factor not only to enable efficient management and to maximize production and profitability, but also for optimum husbandry practices (Leatherland and Cho 1985; Kristiansen et al. 2004; Rowland et al. 2006).

The goal of this study was to evaluate the influence of stocking density and the influence of four commercial diets for marine fish, on growth, condition factor, percent of fish without anomalous pigmentation, and survival of juvenile *A. percula*.

## Material and methods

The experiment was carried out at the Ornamental Aquaculture Laboratory of Polytechnic Institute of Leiria. A total of 530 *Amphiprion percula* juveniles averaging 142.1 ± 2.8 mg and 1.920 ± 0.01 cm (mean ± SEM) were obtained on a local producer of ornamental fish. Clownfish were initially stocked in recirculating system of 4 rectangular tanks in a total volume of 1.2 m^3^ during 3 weeks for acclimatization of laboratory conditions, in a temperature-controlled room 26 – 28°C with lighting at a photoperiod of 14:10 light:dark cycle (Johnston et al. 2003; Olivotto et al. 2011; Dhaneesh et al. 2012a). Water quality was measured weekly, dissolved oxygen, temperature salinity and pH, was measured with a multiparameter meter (YSI; Professional Plus; USA) and total ammonia, nitrite and nitrate was measured by photometric tests using NOVA 60 A Spectroquant® (Merck, Germany). Salinity was maintained at 33 ppt with the addition daily of freshwater using an osmoregulator (TMC,V2 Auto Top Up System, Bristol, UK) that provided automatic compensation of evaporated water with freshwater.

After the acclimatization period, the fish were divided for two experimental designs:

### Trial One – Effect of commercial diets

The research setup of diets trials consisted of a recirculating system of twelve 15-L (30 · 20 · 25 cm, L · W · H) glass aquaria connected to a biological filter, a water pump, UV sterilizer and a sand filter, which was filled with filtered seawater. The flow rate into each aquarium was 45 Lh^−1^. 180 fish were randomly sampled and measured and randomly distributed into twelve tanks at stocking density 1 fishL^−1^ of water (3 replicates for treatment). Fish were feed ad libitum three times daily with four different commercial granulate food with different crude protein levels, 52,5, 48, 41 and 38% (Diet A, B, C and D) during four months. The nutrient composition of the four diets are shown in Table 1. Gross energy content of the diets was calculated on the basis of their crude protein, total fat (ether extract) and nitrogen free extract (NFE) contents using the equivalents of 23.64, 39.54, and 17.15 MJkg^−1^ (Ali et al. 2008). The identities of the products employed are not revealed to make any objective judgment of the findings. To standardize all diets and adapt de pellet size to the fish, all diets were mashed, and after passed thought different mesh- sieves to obtain pellets with 200 to 250 μm.

**Table 1:** **Diets composition expressed as a percentage of dry weight**

**Diets**	**A**	**B**	**C**	**D**
Crude protein	52,5	48,0	41,1	38,1
Crude fat	8,1	8,0	9,0	9,7
Moisture	5,1	10,0	14,5	14,5
Fibre	3,5	4,0,2,4	2,8	
Nitrogen-free extract	24,3	12,0	22,5	24,4
Ash	6,5	18,0	10,5	10,5
Gross energy (MJkg^-1^)	19,8	16,6	17,1	17,0
Gross energy (kcalkg^-1^)	4723,7	3956,5	4091,5	4066,0
P⁄E (g protein MJ^-1^GE)	26,5	29,0	24,0	22,4
P⁄E (mg protein kcal^-1^GE)	111,1	121,3	100,5	93,7

### Trial two – Effect of stocking density

The research setup of stocking density trials consisted of a recirculation system of twelve 17-L (30 · 23 · 25 cm, L · W · H) glass aquaria connected to a biological filter, a water pump, UV sterilizer and a sand filter, which was filled with filtered seawater. The flow rate into each aquarium was 50 Lh^−1^.

330 fish were randomly sampled and measured and randomly distributed into twelve tanks at four different stock densities, 0.5,1, 2 and 3 fishL^−1^ of water (3 replicates for treatment). Fish were feed *ad libitum* three daily with mix of four commercial granulate food (four diets used in experiment 1) during four months.

During the experimental period the water quality was measured, maintained constant with normal parameters for maintaining this specie: DO > 8.0, pH between 8.0 and 8.4 temperature 26 to 28°C, total ammonia and nitrite below 0.5 mgL^−1^ and nitrate < 10 mgL^−1^. At end of both trials all fish were individually anaesthetized with MS-222 (90mgL^−1^) to be sampled, weighed (mg), measured (cm), and the number of fish with miss-bands were recorded.

The influence of the diets and stocking density were evaluated by the mortality, specific growth rate (SGR % length gain day^−1^)^1^ and condition factor (K)^2^.

$$ {}^1\mathrm{S}\mathrm{G}\mathrm{R} = 100\ \left(\mathrm{In}\ \mathrm{L}\mathrm{t}\hbox{-} \mathrm{In}\ \mathrm{L}\mathrm{i}\right)/\mathrm{t} $$

Where Li and Lt, are the initial and final body lengths and the t is time in days.

$$ {}^2\mathrm{K} = 100\ \left(\mathrm{W}/{\mathrm{L}}^3\right) $$

Where W is the final body weight (mg) and L the final body length (cm).

SGR was selected as the most appropriate growth measure as it is relative to initial body parameters and this standardisation best mitigates the effects of varying sizes of fish (Rushworth et al. 2011). In general SGR was calculated in base of weight gains, however generally ornamental species are sold per unit and graded by size rather than by weight, so we decide use fish growth performance in terms of size increment rather than weight. Condition factor is calculated from the relationship between the weight of a fish and its length (Sarkar et al. 2013).

Growth performance parameters are reported as means ± standard error of mean (SEM) of average values per tank of each parameters. All data were checked for normality and homoscedasticity. A one-way analysis of variance (ANOVA) was used to determine significant differences of the effect of the experimental densities and diets (Zar 2009). Post hoc pairwise analysis (Tukey test) was conducted to determine significant differences among experimental combinations. When assumptions (that is, normality and homoscedasticity) were not met, Kruskal–Wallis, followed by Games-Howell test was employed as appropriate (Games and Howell 1976; Kirk 1982). For all statistical tests, the significance level was set at p ≤ 0.05. All calculations were performed with IBM SPSS Statistics 20.

## Results

### Trial one

**Figure 1:**
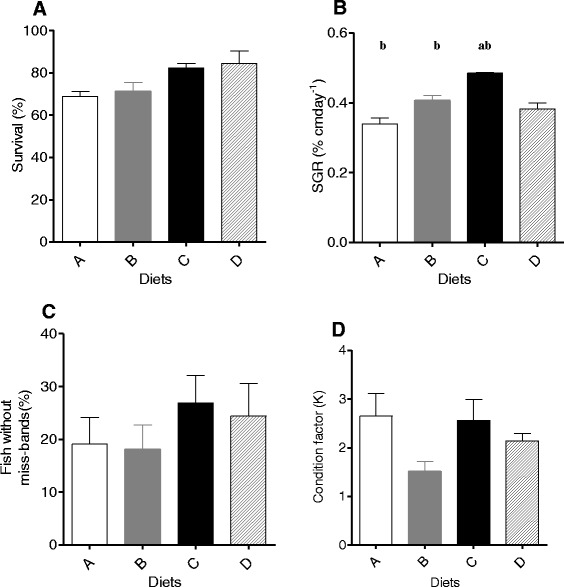
Effect of different diets composition on survival **(A)**, specific growth rate **(B)**, condition factor **(C)** and fish without miss-bands **(D)**. Values are expressed by means of mean ± SEM (n = 3). Lowercase represents significant statistical differences at level p < 0.05: (a) between diet C and other diets; (b) between diet A and diets B and C.

The mean survival reached in groups feeding different diets varied between 68.89 ± 2.22 to 84.44 ± 5.87%, but no statistically differences were obtained (p > 0.05) (Figure 1A). Final lengths varied between 2.865 ± 0.066 to 3.108 ± 0.066 cm and the SGR varied from 0.340 ± 0.016 to 0.485 ± 0.001% cmday^−1^, statistically differences were obtained, the higher SGR was obtained for diet C (ANOVA, F_(3,8)_ = 18.41, p = 0.001) (Figure 1B).

Condition factor c) and fish without miss-bands d) varied between 1.517 ± 0.199 to 2.651 ± 0.47 and 18.03 ± 4.69 to 26.92 ± 5.18, however, no differences were reach between the different commercial diets tested (p > 0.05).

The best SGR was obtained with the diet C, and there was no significance differences in survival, fish without miss-bands or condition factor among diets during the experimental period.

### Trial two

**Figure 2:**
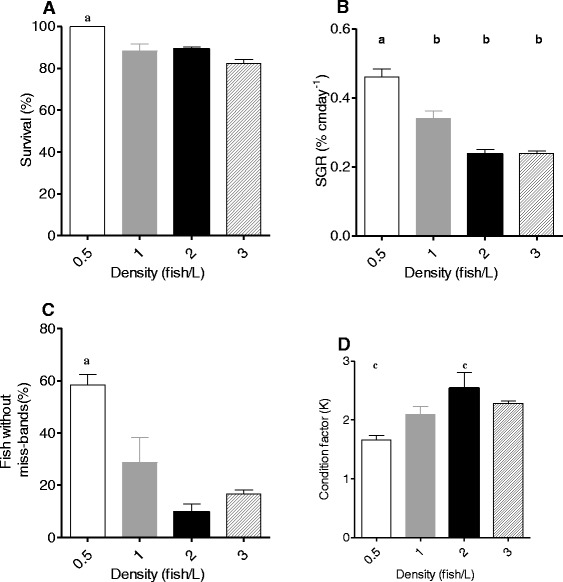
Effect of different stocking density on survival **(A)**, specific growth rate **(B)**, condition factor **(C)** and fish without miss-bands **(D)**. Values are expressed by means of mean ± SEM (n = 3). Lowercase represent statistical significant differences at level p < 0.05: (a) between 0.5 fishL^−1^ and the other stock densities; (b) between 1 fishL^−1^ and 3 fishL^−1^; (c) between 0.5 and 2 fishL^−1^.

The survival varied between 82.5 ± 1.9% to 100%. The high survival rate was obtained for stocking density of 0.5 fishL^−1^ (ANOVA, F_(3,8)_ = 13.240, p = 0.001) (Figure 2A). Final lengths of the fish stocked at a density of 0.5, 1, 2 and 3 fishL^−1^ reached means of 3.350 ± 5 0.06 , 2.977 ± 0.068, 2.633 ± 0.049 and 2.612 ± 0.04 cm respectively.

SGR varied between 0.237 ± 0.013 to 0.459 ± 0.023% cmday^−1^. The best SGR (Figure 2B) was obtained for stocking density of 0.5 fishL^−1^ (ANOVA, F_(3,8)_ = 36.99, p = 0.001). Fish reared at 2 fishL^−1^ obtained the best K 2.537 ± 0.27 (Kruskal-Wallis, K = 8,12,, p = 0.034) (Figure 2C) and the maximum of fish without miss-bands (Figure 2D) were obtained at 0.5 fishL^−1^ (58.33 ± 4.16%) (ANOVA, F_(3,8)_ = 15.28, p = 0.001).

The best survival, SRG and the minimum percent of fish without miss-band was obtained for densities of 0,5 fishL^−1^, however, the best condition factor was obtained with stocking density of 2 fishL^−1^.

## Discussion

Stocking densities and ration size are two important factors affecting fish growth (Hernández et al. 2001; Salas-Leiton et al. 2008; Saoud et al. 2008). In this study, the diets influence the specific growth rate, but not influence the survival, condition factor and fish without miss bands. No relation was observed between malpigmentations and the different types of diets tested. However, other studies demonstrated that polyunsatured fatty acids increase the development of a proper pigmentation (Avella et al. 2007). In case of the halibut and in the turbot, Bell et al. (2003) showed that a 2:1 ratio of DHA:EPA improves de dorsal pigmentation in this species.

In this study the use of diet C improve an increase of more than 0.49% cmday-1, comparatively to other rations tested. All diets under study have different level of crude protein and the best growth parameters was diet C, that have the second lowest level of crude protein (41%), for some species increasing dietary protein can lead to improved fish production, especially in case of carnivorous fish, however the optimal protein requirements differ with fish species, size, water temperature, quality and diet formulation (Ali et al. 2008). Vijayagopal et al. (2008) reported the best for juvenile marine ornamental fish, striped damsel, *Dascyllus aruanus* using ration with crude protein between 360 and 470 g kg, above and below this interval the SGR decreases. In generally 60% of production cost is relatively to the fish feed and protein is the component most expensive, optimizing dietary concentration is essential to increasing the profit of the fish culture, the results achieved with the diet C corresponding to a huge improve on the mass scale production at end of one cycle production (Luo et al. 2004; Ghazala et al. 2011).

Stocking density is an area of particular concern in the welfare of intensively farmed fish (Ashley 2007; Wocher et al. 2011). In this experiment the survival, the specific growth rate and percentage of fish without miss-bands increase with decrease of stocking density.

Ornamental fishes with anomalous pigmentation, namely clownfishes with miss-bands have little demand in the ornamental market and are sold at a lowest prices (Avella et al. 2007). Malpigmentation can caused by several environmental factors such as nutrition, substrate, density and light, but it is unclear the factors that affect malpigmentation, some authors revels that mechanisms of malpighmentation can be associated with stress of stocking density, in the wild olive flounder no phenomens of malpigmentaion are observed but is very common in domesticated flounder that are reared at high densities, (Kang and Kim 2013), our results are consistent with results reported by (Takahashi 1994) that suggested that abnormal pigmentation increases with stocking density.

The effect of stocking density on growth are in line with the view that in many cultured fish species growth is inversely related to stocking density and this is mainly attributed to social interactions, this is most special in clownfish that’s lives in social groups based in size hierarchy (Colleye et al. 2009; Sánchez et al. 2010; Larsen et al. 2012). Clownfish controlling their size and growth rate according with their position in the group hierarchy (Buston 2003; Parmentier et al. 2009). However, the effect of stocking density can varied with the species, adverse effects on growth performance at high stocking densities were observed in juveniles bluegills (*Lepomis macrochirus*), amur sturgeon (*Acipenser schrenckii*), and nile tilapia (*Oreochromis niloticus*) (Yi and Kwei Lin 2001; Anderson et al. 2002; Ni et al. 2014). In contrast, null effects of high densities upon production were reported in juvenile cod (*Ghadus morhua* L.), european sea bass (*Dicentrarchus labrax*) and rainbow trout (*Oncorhynchus mykiss*) (Björnsson and Ólafsdóttir 2006; Lupatsch et al. 2010; McKenzie et al. 2012). Generally an increment of stocking density causes a deterioration of water quality and in poorly conditions fish grow less and die more and some authors use this explanation for the negative effect on growth performance at high stocking densities. Sometimes negative effects of high stocking density on fish growth and survival can be caused by deterioration of water quality, causing the increment of fish metabolites and carbon dioxide with reduction of pH level system (Ruyeta et al. 2007; Hosfeld et al. 2009). Nevertheless in this study the cause of SGR and survival decrease with increase of stocking density cannot be explained through a deterioration of water quality, since all treatments were insert in a same system (Ruyeta et al. 2007; Hosfeld et al. 2009).

The condition factor represents a quantitative indicator of the well-being of fish, and ornamental fish are judged for quality based on appearance and physical attributes (Moorhead and Zeng 2010; Guidelli et al. 2011). High condition factor values appears when fishes are on favourable environmental conditions, by contrast when fishes are in less favourable environmental conditions shows low values (Blackwell et al. 2000). Condition factor in our studies were similar to results obtained by Rushworth et al. (2011) in the culture of juvenile wide-band anemonefish (*Amphiprion latezonatus*) at different temperatures. The results presented here clearly showed that in all treatments fish were on good conditions.

Our results of survival and condition factor were similar to results obtained by (Johnston et al. (2003)) that studied the effect of different of ration size and feeding frequency on growth of juvenile *Amphiprion percula*.

Generally, the results suggest that stocking density have more effects comparing with effects of diets on specific growth rate, condition factor, the miss-band and survival of juvenile percula clownfish.

## Conclusions

The production in captivity of ornamental fishes is the solution for to guarantee a long term sustainable trade and the way to preserve marine biodiversity.

This study defined optimal conditions helped for the production of this tropical coral reef ornamental fish in captivity, concerning anemonefishes husbandry in terms of survival and quality of fish pigmentation. It is believed that a compound diet of 41% protein would provide nutrients that will ensure optimal growth of *A. percula*, stocking density are critical and relevant point to enhance the captive production of tropical marine species for the aquarium trade. However the stock density that fish showed lowest malpigmentation is very lower to guarantee economic viability of mass production of this species, so more studies are necessary to turned profitable the mass production of this species.
